# Evaluation of flow of chorioretinal capillaries in healthy black and white subjects using optical coherence tomography angiography

**DOI:** 10.1038/s41598-021-01380-6

**Published:** 2021-11-08

**Authors:** Nathalie Massamba, Anna G. Mackin, Lindsay Y. Chun, Sarah Rodriguez, Rose C. Dimitroyannis, Bahram Bodaghi, Seenu M. Hariprasad, Dimitra Skondra

**Affiliations:** 1 Department of Ophthalmology and Visual Science, The University of Chicago, 5841 S. Maryland Avenue, S426m MC2114, Chicago, IL 60637 USA; 2 Department of Ophthalmology, Handicap, and Vision, Pitie Salpetriere Hospital, Sorbonne University, Paris, France; 3 J. Terry Ernest Ocular Imaging Center, The University of Chicago, Chicago, IL USA

**Keywords:** Vision disorders, Diagnostic markers

## Abstract

This study compared macular capillary parameters between healthy black and white subjects using optical coherence tomography angiography (OCTA). We measured vessel density (VD) of superficial (SCP), intermediate (ICP), and deep (DCP) capillary plexuses and choriocapillaris blood flow area (BFA) of the fovea, parafovea and total 3 mm-diameter circular area centered on the fovea, as well as the foveal avascular zone (FAZ) parameters, controlling for axial length. Black subjects had lower foveal and parafoveal VD in the SCP (*p* = 0.043 and *p* = 0.014) and the ICP (*p* = 0.014 and *p* = 0.002). In the DCP, black subjects had a trend toward lower foveal and parafoveal VD. Black subjects had decreased choriocapillaris BFA in the total 3 mm area (*p* = 0.011) and the parafovea (*p* = 0.033), larger FAZ area (*p* = 0.006) and perimeter (*p* = 0.014), and a higher capillary density in a 300 μm wide region around the FAZ (FD-300) (*p* = 0.001). There was no significant difference in FAZ acircularity index. To our knowledge, this is the first report analyzing the three distinct retinal capillary plexuses and identifying differing baseline VD, choriocapillaris and FAZ parameters in healthy young black compared to white subjects. Larger studies are needed to validate these findings and better understand racial differences in vulnerability to ocular diseases.

## Introduction

Morphological studies in humans and animal models have demonstrated the presence of three distinct capillary plexuses in the macula: superficial capillary plexus (SCP), intermediate capillary plexus (ICP), and deep capillary plexus (DCP). It was shown that the inner plexiform and the outer plexiform layers of the retina, supplied by the ICP and the DCP respectively, are particularly vulnerable to ischemic injury due to these layers’ high metabolic activity^1–3^. The visualization of ICP in particular was difficult in the past because of this layer’s thinness, and imaging artifacts^4,5^. Optical coherence tomography angiography (OCTA), rapid and non-invasive imaging modality optimal for assessing retinal and choroidal vascular parameters^6–8^, has allowed for the first time to visualize and analyze the three distinct retinal capillary plexuses in vivo with the help of novel projection artifact removal (PAR) software and improved segmentation^9,10^. The ability to evaluate the microvasculature of all three capillary layers in healthy and diseased eyes may significantly enrich our understanding of retinal disorders.

Diabetic retinopathy and retinal vein occlusion, associated with ischemic retinal injury, have a more aggressive presentation in black populations^11–13^. Glaucoma, recently shown to be associated with decreased retinal capillary perfusion within the superficial capillary plexus^14^, is known to be more prevalent and more severe in black as compared to white people^15–19^. Better understanding of baseline characteristics of the three retinal capillary plexuses and the choriocapillaris in black as compared to white subjects may contribute to our understanding of the role of race as a risk factor in retinal vascular diseases and glaucoma. Our study aims at comparing baseline retinal vascular parameters including vessel density (VD) of SCP, ICP and DCP, choriocapillaris blood flow area (BFA), as well as characteristics of foveal avascular zone (FAZ) in healthy young black and white individuals using OCTA, in an effort to identify any differences that may in turn have clinical implications.

## Subjects and methods

### Study design

This prospective cross-sectional study was conducted between September 2017 and June 2019 in adherence to the tenets of the Declaration of Helsinki and the Health Insurance Portability and Accountability Act regulations. The study was approved by the Institutional Review Board (IRB) of the University of Chicago (IRB #18–1174, #17–0170). All subjects provided written informed consent.

### Participants

The study included black and white adult subjects of 18 years of age or older, non-smokers, without history of any medical or ocular diseases. Some of these patients and their OCTA scans were included in our previous study^20^; an additional group of 11 healthy patients (19 eyes) was also evaluated. Subjects were recruited among Eye Clinic patients, University of Chicago students and members of the surrounding local community. Demographic information was collected including age, sex, race, smoking history, medical and ocular history. To determine racial identity, subjects self-identified as black, white, East Asian, Southeast Asian, Indian, Native American, Latino, mixed, or other. Subjects who selected categories other than “black” or “white”, or selected multiple racial categories were excluded. The following subjects were excluded: current smokers, former smokers with greater than 100-cigarette lifetime smoking history, subjects with history of systemic medical conditions requiring medications or interventions, subjects with any ocular conditions (such as glaucoma, retinal disorders, uveitis, cataracts, blindness), subjects with other conditions that could potentially affect the retinal microvasculature or prevent adequate OCTA imaging, and subjects showing abnormalities on OCTA imaging. The subjects' spherical equivalent (SEq, a sum of spherical power and half of cylinder power) was calculated by using refractive error as measured by autorefraction (KR-8000 AutoKerato Refractometer, Topcon Omni Systems, Oakland, NJ, USA), lensometry (Topcon CL-2000 Computerized Lensometer, Topcon Omni Systems, Oakland, NJ, USA), or reported in most recent glasses prescription. Eyes with significant refractive error (myopia of 5 diopters (D) or more, or hyperopia of 3 D or more) were excluded from this study. Axial length was measured using TopCon Aladdin HW3.0 software Biometer with corneal Topography.

### OCTA image acquisition

OCTA imaging was performed with the Optovue RTVue XR Avanti spectral-domain instrument (Optovue Inc, Fremont, CA, USA Version 2016.2.0.35) with phase 7 AngioVue software. Images were taken using 840 nm light source, 45 nm bandwidth, and an A-scan rate of 70,000 scans per second. Each scan consisted of 608 B-frames, composed of a set of 304 A-lines acquired 2 times at each of the 304 raster positions. The scanning area used in this study was 3 × 3 mm, centered on the fovea. The software included the 3D Projection Artifact Removal (PAR) algorithm^9^. Images with Signal Strength Index (SSI) below 70 were excluded. The Quality Index (QI) (range 1 to 10) accounted for the signal strength and 2 additional factors: motion artifacts and image sharpness. Only images with QI of 7 or greater (manufacturer's recommendation) and without large movement or shadow artifacts were considered for further analysis.

### Analyzing three retinal capillary plexuses and the FAZ

**Figure 1:**
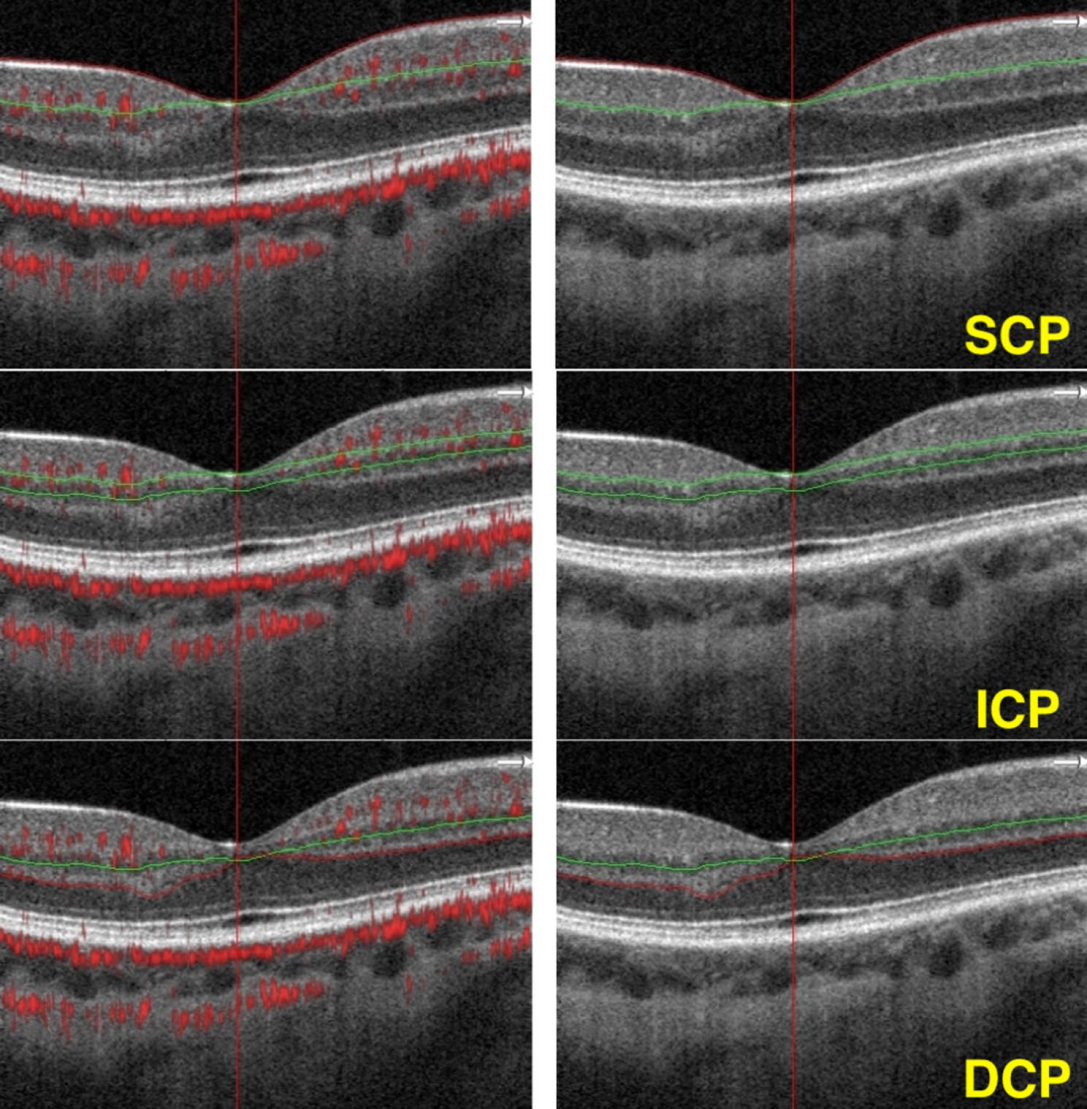
Optical coherence tomography B-scans showing the retinal segmentation in different capillary plexuses. Superficial capillary plexus (SCP): boundaries segmented from the internal limiting membrane (red line) to 10 μm above the inner plexiform layer (green line). Intermediate capillary plexus (ICP): boundaries (green lines) segmented from 10 μm above to 30 μm below the inner plexiform layer. Deep capillary plexus (DCP): boundaries segmented from 30 μm below the inner plexiform layer (green line) to 10 μm below the outer plexiform layer (red line). All images come from the same eye of a single subject. Images were created using Preview Version 10.1 (944.6.16.1) (https://support.apple.com/guide/preview/welcome/mac), and Fiji Version 2.0.0-rc-69/1.52p (https://imagej.net/software/fiji/).

We used customized segmentation for the SCP, ICP, and DCP in our analysis, as described by Nesper et al.^21^.The SCP boundaries were defined from the internal limiting membrane (ILM) to 10 um above the inner plexiform layer (IPL) to encompass the nerve fiber and ganglion cell layers. The ICP was defined from 10 um above to 30 um below the IPL to encompass the IPL. The DCP was defined from 30 um below the IPL to 10 um below the outer plexiform layer (OPL) to encompass the OPL (Fig. 1).

### Vessel density measurements and FAZ parameters

**Figure 2:**
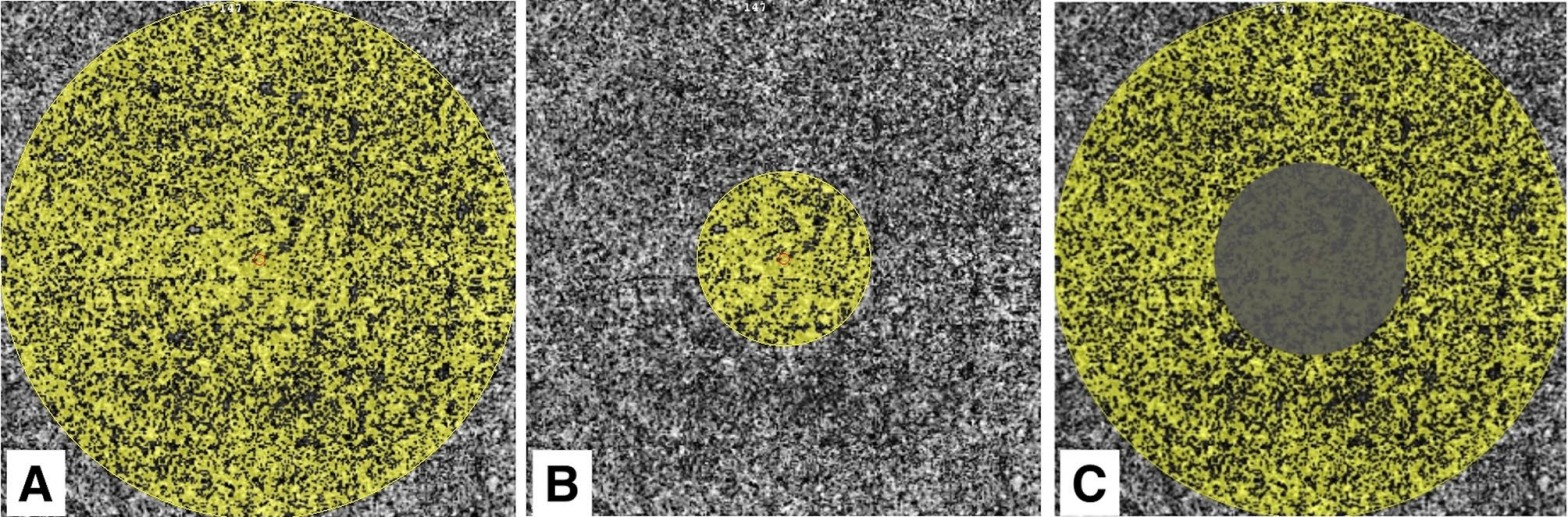
Definition of the macula/fovea/parafovea areas from en face OCTA images using OCTA AngioAnalytics software. En face OCTA images representing the areas (shaded in yellow) corresponding to (**A**) the 3 mm-diameter macular area, (**B**) the central 1 mm-diameter circular area of the fovea, (**C**) the parafovea. Images were created using Preview Version 10.1 (944.6.16.1) (https://support.apple.com/guide/preview/welcome/mac), and Fiji Version 2.0.0-rc-69/1.52p (https://imagej.net/software/fiji/).

**Figure 3:**
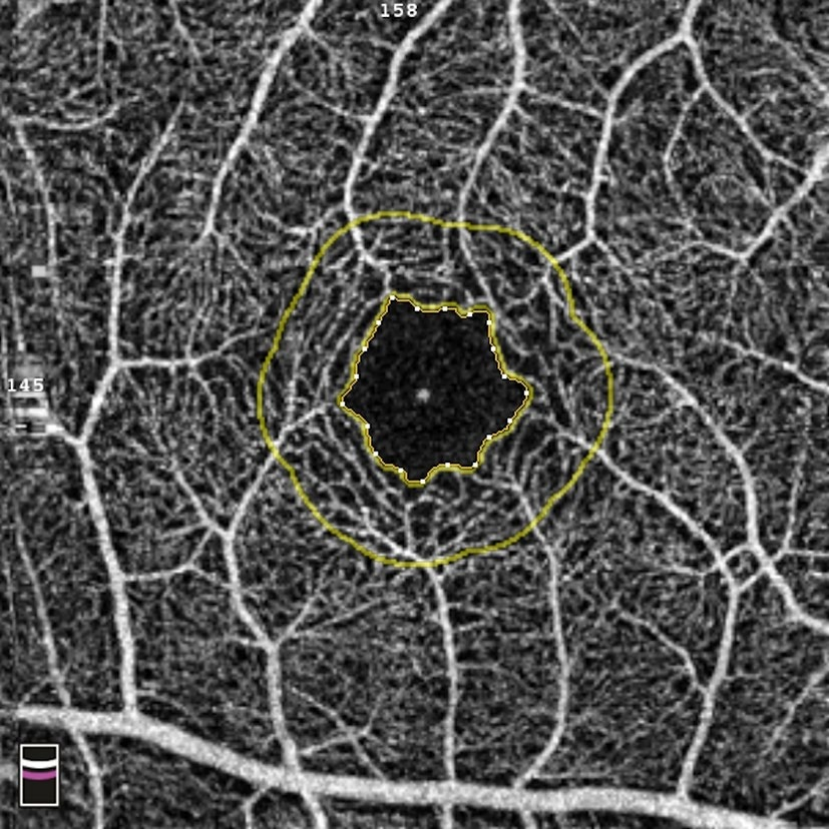
Definition of the foveal avascular zone (FAZ). Optical coherence tomography angiography map showing the contour of the FAZ (inner yellow ring), and a 300 μm wide area surrounding the FAZ (FD-300) (outer yellow ring). Images were created using Preview Version 10.1 (944.6.16.1) (https://support.apple.com/guide/preview/welcome/mac).

Using the AngioAnalytics software, we calculated VD as the percentage of the image occupied by retinal blood vessels within the SCP, ICP, and DCP layers in the fovea, parafovea and a 3-mm-diameter circle centered on the fovea. The foveal area was defined as a 1-mm-diameter circle centered on the fovea. The parafoveal area was defined as a ring from the edge of the foveal circle to the edge of a 3-mm-diameter circle centered on the fovea (Fig. 2). The FAZ size (in mm^2^) was automatically calculated by the AngioAnalytics software using the nonflow area measurement tool. FAZ perimeter (mm) was defined as the length of the contour demarcating the FAZ. FAZ acircularity index (AI) was calculated as the ratio of the FAZ perimeter to the perimeter of a circle with an equal area, AI = FAZ Perimeter/√(4 × π × Area)^13^. We also recorded FD-300, a parameter calculated by the AngioAnalytics software as retinal capillary density from ILM to OPL in a 300 μm wide region around the FAZ (Fig. 3).

### Analysis of the choriocapillaris blood flow area

Blood flow area (BFA), the percentage of a retinal image occupied by pixels corresponding to blood vessels, was calculated automatically after the image capture process by AngioAnalytics software^15^. In this study we obtained choriocapillaris BFA within 1 mm- and 3 mm-diameter circular zones centered manually on the fovea. The choriocapillaris BFA in the parafoveal region was determined by subtracting the BFA of the 1 mm-diameter circular area from the BFA of the 3 mm-diameter circular area centered on the fovea (Fig. 2).

### Statistical analysis

Statistical analysis was performed using Stata 15 (StataCorp LP, College Station, TX). Percentages or mean values with standard deviation were reported, using frequency weighting to account for the correlation between the right and left eyes of the same subject. For baseline data, P values were calculated using Pearson's chi-squared test for categorical variables and the Wilcoxon rank-sum test for continuous variables. To evaluate the association between race and mean macular capillary parameters, a mixed-effects linear regression model was used, accounting for 2 eyes of the same subject, and adjusting for axial length. A *p*-value of < 0.05 was considered statistically significant.

## Results

One hundred twelve eyes (54 right and 58 left) of 58 subjects were included in this study. Twenty-nine subjects self-identified as black (mean age 27.5 + /− 4.9 years, 55% male, 45% female) and 29 subjects self-identified as white (mean age 27.8 + /− 4.2 years, 45% male, 55% female). There were no significant differences in the demographics, refractive error, or axial length parameters between the two racial groups (Table 1). There were no significant differences in either the QI (mean QI 8.4 ± 0.98 in black vs 8.6 ± 0.6 in white subjects, *p* = 0.77) or the SSI (71.9 ± 7.4 in black vs 72.3 ± 8 in white subjects, *p* = 0.81).

**Table 1:** Demographics and baseline clinical characteristics.

	White subjects	Black subjects	*P*-value
Sex			0.38
Male (%)	13 (45%)	16 (55%)	
Female (%)	16 (55%)	13 (45%)	
Age, years	27.8 ± 4.2	27.5 ± 4.9	0.55
Axial Length (mm)	23.8 ± 1.5	24.4 ± 1.3	0.08
Spherical equivalent, diopters	− 0.9 ± 1.4	− 1.15 ± 1.7	0.94
Image quality index	8.6 ± 0.60	8.4 ± 0.98	0.77
Signal strength index	72.3 ± 8	71.9 ± 7.4	0.81

Data are presented as mean ± standard deviation. For baseline data, P values were calculated using Pearson's chi-squared test for categorical variables and the Wilcoxon rank-sum test for continuous variables.

Sixteen patients (10 black, 6 white; 32 eyes total) did not have axial length measurements available for analysis. We performed mixed-effects linear regression model analysis of all subjects controlling for two eyes of the same subject and controlling for axial length while excluding the subjects without available axial length measurements. Most of the relationships between macular capillary parameters and race were consistent across these two analyses (Table 2).

**Table 2:** Mixed-effects linear regression analysis of the association between race and mean macular capillary parameters.

Macular capillary parameter	Regression coefficient	*P* value	Regression coefficient*	P* value
**SCP VD (%)**				
Fovea	2.53 (0.37–5.04)	0.047	2.81 (0.084–5.54)	0.043
Parafovea	1.71 (0.45–2.97)	0.008	1.37 (0.27–2.47)	0.014
Total area of fovea and parafovea	0.98 (− 0.31–2.26)	0.136	0.66 (− 0.54–1.86)	0.284
**ICP VD (%)**				
Fovea	4.21 (1.24–7.18)	0.005	3.96 (0.82–7.11)	0.014
Parafovea	2.24 (0.69–3.78)	0.005	2.64 (0.98–4.30)	0.002
Total area of fovea and parafovea	1.26 (− 0.23–2.76)	0.097	1.54 (0.01–3.06)	0.048
**DCP VD (%)**				
Fovea	1.67 (− 0.91–4.26)	0.205	2.28 (− 0.41–4.96)	0.096
Parafovea	1.25 (− 1.28–3.78)	0.332	2.25 (− 0.42–4.92)	0.098
Total area of fovea and parafovea	0.86 (− 1.48–3.19)	0.473	1.54 (− 0.98–4.07)	0.23
**FAZ Area (mm**^**2**^**)**	− 0.10 (− 0.16–(− 0.05))	< 0.001	− 0.07 (− 0.12–(− 0.02)	0.006
**FAZ Perimeter (mm)**	− 0.29 (− 0.46–(− 0.12))	0.001	− 0.23 (− 0.41–(− 0.47))	0.014
**FAZ Acircularity Index**	0.035 (0.008–0.061)	0.01	0.023 (− 0.002–0.49)	0.076
**FD-300**	− 2.64 (− 4.02–(− 1.23))	< 0.001	− 2.34 (− 3.71–(− 0.97))	0.001
**Choriocapillaris BFA (%)**				
Fovea	1.2 (0.06–2.3)	0.039	1.1 (− 0.10–2.3)	0.073
Parafovea	2.4 (0.46–4.4)	0.016	2.4 (0.19–4.6)	0.033
Total area of fovea and parafovea	1.5 (0.47–2.5)	0.004	1.5 (0.34–2.6)	0.011

Regression Coefficient and P value accounting for 2 eyes of the same subject. Regression Coefficient* and P* value accounting for 2 eyes of the same subject and adjusting for axial length. VD = vessel density. SCP = superficial capillary plexus. ICP = intermediate capillary plexus. DCP = deep capillary plexus. FAZ = foveal avascular zone. FD-300 = capillary density in a 300 μm wide region around the FAZ. BFA = blood flow area.

Vessel density values in all three capillary plexuses are reported in Table 3. Within the SCP, black subjects showed significantly lower VD values in the fovea (mean VD 18.2 ± 6.01% in black vs 20.7 ± 3.90% in white subjects, *p* = 0.043) and the parafovea (mean VD 49.9 ± 2.75% in black vs 51.2 ± 2.38% in white subjects, *p* = 0.014). There was a trend toward lower VD within the SCP of the total area encompassing the fovea and parafovea in black as compared to white subjects (mean VD 47.1 ± 2.73% in black vs 47.8 ± 2.30% in white subjects, *p* = 0.284), however it did not reach statistical significance. In the ICP, black subjects showed significantly lower VD in the fovea (mean VD 26.5 ± 8.61 in black vs 30.4 ± 7.55% in white subjects, *p* = 0.014), the parafovea (mean VD 45.1 ± 2.81% in black vs 47.2 ± 3.11% in white subjects, *p* = 0.002), and the total area encompassing the fovea and parafovea (mean VD 43.4 ± 2.70% in black vs 44.9 ± 2.53% in white subjects, *p* = 0.048). Within the DCP, there was a trend toward lower VD among black subjects in the fovea (mean VD 20.3 ± 7.03% in black vs 21.7 ± 5.72% in white subjects, *p* = 0.096), the parafovea (mean VD 40.0 ± 6.18% in black vs 40.7 ± 5.14% in white subjects, *p* = 0.098), and the total area encompassing the fovea and parafovea (mean VD 37.5 ± 5.26% in black vs 38.1 ± 5.26% in white subjects, *p* = 0.23) however it did not reach statistical significance.

**Table 3:** Macular capillary parameters measured using optical coherence tomography angiography in white and black subjects.

Macular capillary parameter	White subjects	Black subjects	P* value
**SCP VD (%)**			
Fovea	20.7 ± 3.90	18.2 ± 6.01	0.043
Parafovea	51.2 ± 2.38	49.9 ± 2.75	0.014
Total Area of Fovea and Parafovea	47.8 ± 2.30	47.1 + 2.73	0.284
**ICP VD (%)**			
Fovea	30.4 ± 7.55	26.5 ± 8.61	0.014
Parafovea	47.2 ± 3.11	45.1 ± 2.81	0.002
Total Area of Fovea and Parafovea	44.9 ± 2.53	43.4 ± 2.70	0.048
**DCP VD (%)**			
Fovea	21.7 ± 5.72	20.3 ± 7.03	0.096
Parafovea	40.7 ± 5.14	40.0 ± 6.18	0.098
Total Area of Fovea and Parafovea	38.1 ± 5.26	37.5 ± 5.26	0.23
**FAZ Area (mm**^**2**^**)**	0.221 ± 0.07	0.332 ± 0.12	0.006
**FAZ Perimeter (mm)**	1.91 ± 0.30	2.22 ± 0.38	0.014
**FAZ Acircularity Index**	1.15 ± 0.080	1.11 ± 0.036	0.076
**FD-300**	50.3 ± 3.20	53.3 ± 2.84	0.001
**Choriocapillaris BFA (%)**			
Fovea	62.5 ± 1.6	61.0 ± 3.5	0.073
Parafovea	57.3 ± 4.3	54.7 ± 3.3	0.033
Total area of fovea and parafovea	66.5 ± 1.8	64.9 ± 2.9	0.011

Data provided show the results of a mixed effects regression correlating race and macular capillary parameters, adjusting for 2 eyes of the same patient and axial length. Data are provided as mean ± standard deviation. Fovea refers to the central 1-mm diameter circular area. Parafovea refers to the 3-mm diameter area excluding the central 1-mm diameter. P* value calculated controlling for 2 eyes of the same patient, and axial length. VD = vessel density. SCP = superficial capillary plexus. ICP = intermediate capillary plexus. DCP = deep capillary plexus. FAZ = foveal avascular zone. FD-300 = capillary density in a 300 μm wide region around the FAZ. BFA = blood flow area.

Foveal avascular zone parameters are reported in Table 3. Black subjects had significantly larger FAZ area (mean FAZ area 0.332 ± 0.120 mm^2^ in black vs 0.221 ± 0.705 mm^2^ in white subjects, *p* = 0.006) and larger FAZ perimeter than white subjects (mean FAZ perimeter 2.22 ± 0.38 mm in black vs 1.91 ± 0.30 mm in white subjects, *p* = 0.014). There was no significant difference in FAZ AI between black and white subjects (mean FAZ AI 1.11 ± 0.036 in black vs 1.15 ± 0.080 in white subjects, *p* = 0.076). Vessel density in FD-300 was significantly higher in black as compared to white subjects (FD 53.3 ± 2.84 in black vs 50.3 ± 3.20 in white subjects, *p* = 0.001).

BFA in the choriocapillaris (Table 3) was significantly lower in black as compared to white subjects in the parafovea (BFA 59.42% ± 1.1 in black vs 61.16% ± 1.3 in white subjects, *p* < 0.001) and the total 3 mm-diameter circular area centered on the fovea (BFA 60.85% ± 1.1 in black vs BFA 62.78% ± 1.9 in white subjects, *p* > 0.001). There was a trend toward lower BFA in the fovea in black subjects, however this trend did not reach statistical significance (BFA 60.14% ± 1.7 in black vs 60.81% ± 2 in white subjects, *p* = 0.11).

## Discussion

To date little is known about possible baseline retinal vascular differences across races. To our knowledge, this is the first study evaluating the three retinal capillary plexuses in the normal eyes of black and white young healthy subjects using the latest OCTA software. Our study showed significantly lower VD in the SCP and ICP in the fovea and parafovea in black subjects, as well as a trend toward lower foveal and parafoveal VD in the DCP. We also noted significantly larger FAZ area and perimeter in black subjects. These findings may point toward innate baseline differences in the vascular anatomy of black and white subjects, which may contribute to differing vulnerability to retinal vascular diseases and glaucoma in black patients along with other genetic and socio-economical risk factors previously suggested.

Our earlier study comparing macular capillary parameters in young healthy black and white subjects utilized a previous version of the software segmentation algorithm, which merged the ICP together with the SCP and DCP^20^. The major contribution of this current work is the separate analysis of the three distinct retinal capillary plexuses. Unlike our earlier study, which showed a significantly lower macular VD within the DCP, and a trend toward lower macular VD within the SCP in black subjects, this study showed a significantly lower macular VD within the SCP and ICP, and a trend toward lower VD within the DCP. We believe that the deviation between our earlier and current results may arise from the differences in VD quantification stemming from improved software for artifact removal and different segmentation slabs, rather than distinctly different anatomical patterns.

Upon review of literature, we have noted that in most reports of OCTA parameters in retinal vascular diseases the race of subjects is not specified, which may introduce a confounding bias and affect data interpretation. Our findings of baseline differences in macular capillary parameters among young healthy black and white subjects suggest that these OCTA metrics may vary in populations in ways that are not related to disease. It is important to consider race when analyzing retinal vasculature among different study groups and creating normative databases involving OCTA parameters. Furthermore, as our understanding of the chorioretinal vascular anatomy and physiology evolves with the help of novel imaging modalities, we may choose to select biomarkers that are less susceptible to variation by race in the healthy population.

Black patients with type 2 diabetes mellitus are known to have significantly higher rates of proliferative diabetic retinopathy and diabetic macular edema, and are more likely to experience vision loss and blindness as a result of diabetic retinopathy as compared to non-hispanic white patients^22–25^. In addition, black patients are more susceptible to the retinal vascular effects of hypertension^22^. Furthermore, among patients with retinal vein occlusions, black patients have more severe visual impairment and require more aggressive treatment as compared to white patients^26–29^. Better understanding of baseline retinal vascular parameters across races is necessary to understand and address this difference in vulnerability to the damaging effects of sustained hyperglycemia and hypertension.

Several studies analyzing microvascular parameters of the SCP, ICP and DCP in patients with diabetic retinopathy found a significant reduction in vessel density across all three distinct capillary plexuses^30^, with the degree of vessel density reduction corresponding to the severity of diabetic retinopathy and associated vision loss^30–36^. Recently, the important role of ICP has been distinctly demonstrated with significantly decreased ICP vessel density, perfusion, and blood flow in patients with worsening DR^9,31,37^. Similarly, a significant decrease in macular vessel density within the SCP, ICP and DCP has been demonstrated in patients with BRVO^38^, while the extent of vessel density loss has been shown to correlate with the degree of vision loss following resolution of macular edema^39,40^. VD has been suggested as a potential biomarker of retinal vascular diseases; however there may be a difference in baseline VD within the three distinct capillary plexuses across races as shown in our study. Thus, race needs to be considered as a possible confounder when analyzing macular capillary density parameters in retinal vascular diseases. Choriocapillaris vessel density in the foveal, parafoveal and perifoveal areas has also been shown to decrease in DR and CRVO in association with ischemic damage^41,42^. Our study showed decreased choriocapillaris BFA in healthy black subjects; this finding needs further investigation in larger age-varied populations, since choriocapillaris BFA may have a role in susceptibility to ischemic damage. Considering that decreased vessel density and increased flow voids in the choriocapillaris have been associated with the development and progression of age-related macular degeneration (AMD), our finding of decreased choriocapillaris BFA in healthy young black subjects is interesting in light of the known lower risk of AMD among black people^43,44^. Differential pigment levels among white and black subjects may lead to different levels of imaging artifact, possibly affecting our ability to uniformly quantify the choriocapillaris. Another explanation could be that increased melanin content may affect choriocapillary vasculogenesis, as some preclinical studies have shown that melanin may have antiangiogenic properties that theoretically could affect choriocapillary density and decrease the risk of AMD^45^.

FAZ abnormalities on OCTA imaging have also been associated with retinal vascular diseases. Diabetic retinopathy is associated with enlargement of the FAZ, greater FAZ perimeter, and increased FAZ acircularity index^13^. FAZ enlargement may even represent a pre-symptomatic sign before the clinical onset of diabetic retinopathy^46,47^. Retinal vein occlusions have been associated with enlarged FAZ, increased FAZ acircularity index, a more tortuous pathological FAZ border, and show a negative correlation between visual acuity and FAZ area in patients without macular edema^42,48–50^. Our results indicate a larger FAZ area and perimeter in young healthy black subjects as compared to white subjects. Further validation of this variation is necessary, since baseline differences in FAZ across races may not only affect the validity of this parameter when assessing disease severity, but may also suggest a potential mechanism of greater susceptibility to microvascular changes associated with retinal vascular diseases. The FD-300 value complimentary to other FAZ metrics has been shown to decrease with age in all plexuses and sectors of the macula, and decrease in diabetic retinopathy^51,52^. Our results showed a significantly higher FD-300 density in black as compared to white subjects. This relationship may represent a compensatory mechanism, where the greater capillary density around the foveal avascular zone may compensate for the larger avascular zone and differences in foveal pit morphology in healthy black patients^53^. More studies in larger populations including subjects of different races are necessary to further evaluate FD-300 and its significance.

Recent studies have shown that changes in SCP, which supplies the ganglion cell complex, may play a role in the pathogenesis of open angle glaucoma as well. OCTA imaging studies have shown that patients with open angle glaucoma have a decreased vessel density and a larger area of capillary dropout within the SCP^14,54–56^. Furthermore, increased patient age, a known risk factor for glaucoma development, is shown to be associated with decreased retinal capillary density, however it is unknown whether this trend is different in black as compared to white patients^57,58^. It is unclear whether reduced macular capillary perfusion is contributing to ganglion cell loss in glaucoma, or develops as a consequence of reduced metabolic and vascular demand of already damaged ganglion cells^59^. Our findings of lower baseline vessel density within SCP and ICP in black subjects indicate that race may be a confounder when analyzing the role of superficial retinal vascular complex perfusion in glaucoma development.

The limitations of our study include a small population size, participation of a single clinical site, and engagement of a single image reader. Since our study included only young healthy subjects, these results may not be generalizable to older populations and patients with comorbidities. Our analysis of the chorioretinal capillary parameters is limited by the 3 × 3 mm scan acquisition. Further studies analyzing larger scanned areas can contribute more insights into chorioretinal capillary anatomy and physiology. Additionally, our study utilizes a manual segmentation algorithm, which is limited in its execution by the resolution of the acquired OCTA images and the AngioAnalytics software. The strengths of the study include recruitment of a well-matched patient cohort, and utilization of the latest OCTA software with projection artifact removal allowing analysis of the distinct capillary plexuses.

## Conclusions

In conclusion, this study demonstrates decreased vessel density in SCP and ICP, larger FAZ size and perimeter, and decreased choriocapillaris BFA in young healthy black as compared to white subjects. OCTA is a useful imaging modality to study the role of macular microvascular network heterogeneity among racial groups. Further studies including age-varied subjects are needed to validate these macular microvascular differences in larger populations and elucidate any potential clinical associations.
